# Unexpected events during survey design and trust in the police: a systematic review

**DOI:** 10.1007/s11292-022-09508-y

**Published:** 2022-06-15

**Authors:** Christof Nägel, Amy E. Nivette

**Affiliations:** Department of Sociology, Utrecht University, Utrecht, The Netherlands

**Keywords:** Causal inference, Trust in police, Unexpected event, Ignorability, Excludability

## Abstract

**Objectives:**

The current review has two aims: (1) to synthesize the impact of unexpected events on trust in police across different contexts and types of events, and (2) to evaluate the methodological characteristics of each study with attention to the assumptions for causal inference.

**Methods:**

We conducted a pre-registered narrative systematic review on 12 independent studies.

**Results:**

Studies closely adhering to causal inference assumption checks (i.e., excludability and ignorability) find significant changes in trust in police following incidents of police (non) violence and protest. Still, excludability is assessed and addressed less rigorously than ignorability in the included studies.

**Conclusion:**

Regarding the procedural justice framework, this provides some causal evidence that vicarious (positive and negative) experiences can shape short-term assessments of public trust in police. We furthermore highlight issues related to design and power, statistical conclusion validity, and the evaluation of assumptions to detect threats to internal validity.

**Supplementary Information:**

The online version contains supplementary material available at 10.1007/s11292-022-09508-y.

## Introduction

A great deal of research on perceptions of police tends to be based on cross-sectional correlational studies (Nagin and Telep 2017; Walters and Bolger 2019). While there are a growing number of Randomized Controlled Trials (RCTs), often considered the “Gold Standard” of social scientific research, these studies tend to lack external validity or “policy transfer” (Sampson 2010). Natural experiments, and the use of instrumental variables in general, can offer the opportunity to address some of the limitations of cross-sectional and RCT studies (Angrist 2006). Aside from increasing external validity of research findings (Meyer, 1995), natural experiments might help to more adequately address issues related to simultaneity, measurement error, and selection bias (Bushway and Apel 2010). Furthermore, some obvious ethical problems arise when researching sensitive topics such as the effects of insecurity, political violence, and police abuse on public perceptions of policing in a real-life experimental setting. For example, regarding procedural justice theory, there are clear limits to the manipulation of the displayed (un-)fairness in personal interactions with police officers in survey experiments or even a RCT. In a natural experiment, however, actual events can be used as quasi-experimental stimuli for vicarious experiences, which alleviates some of the ethical problems mentioned above and might even increase internal validity given that the causal inference assumptions are adequately addressed.

Muñoz et al. (2020) have described a special type of natural experiment that may prove particularly important for studying high-profile events and their implications for police legitimacy and trust in the police: the Unexpected Event During Survey Design (UESD). This identification strategy relies on the timing of the interview as the instrumental variable, as it affects the outcome of interest through the occurrence of an unexpected event. This research design has gained prominence through studies that examine the effect of terrorist attacks on attitudes toward ethnic minorities (Bove et al. 2021; Legewie 2013; Nussio et al. 2019).

Within criminology, interest has grown in assessing the impact of unexpected events on public attitudes toward the police (Kochel (2019); Kochel and Skogan (2021); Nägel and Lutter (2021); Reny and Newman (2021); Thomassen et al. (2014)). These events can be considered “vicarious experiences” (Weitzer 2002), depending on the media salience, police reactions, and societal context (Cheng 2021; O’Brien et al. 2020), rendering them appropriate quasi-experimental “manipulations” to study the dynamics of public perceptions of police within the UESD setup. For example, following the death of Michael Brown in Ferguson, Missouri, questions arose as to what extent this incident of police violence mobilized and changed attitudes toward the police (Kochel 2019). Previous research has so far assessed a wide range of events, including incidents involving police actions (e.g., violence, misconduct, policing peaceful protests, Nägel and Lutter 2021; Oglesby-Neal et al. 2019; Reny and Newman 2021; White et al. 2018; Zoorob 2020) as well as large-scale global or societal events (e.g., terrorist attacks, public health crises, Dinesen and Jaeger 2013; Fenn and Brunton-Smith 2021; LaFree and Adamczyk 2017; Sibley et al. 2020). It is important to understand how these events influence trust in police since trust has been associated with cooperation and compliance (Tyler 2006; Tyler and Huo 2002), criminal behavior (Dawson 2018; Eisner and Nivette 2013), trust in the state itself (Jeong and Han 2020), and social polarization in general (Jefferis et al. 1997; Kochel 2019; Nägel and Lutter 2021; Reny and Newman 2021). Accordingly, there is a strong argument that “[s]tudies that are able to capture the dynamic of cleavages and their consequences for police legitimacy in such instances are important” (Roché and Roux 2017, p. 16). However, an appropriate summary of research findings in this strand of research is still lacking, and more importantly, there is little guidance for researchers striving to apply this particular design within the field of criminology.

Although UESD can be beneficial for assessing the impact of high-profile incidents on public opinion, there are important practical and methodological conditions that must be met in order to reliably estimate causal effects (Muñoz et al. 2020). One practical requirement is that the timing of unexpected police events overlaps with survey fieldwork that includes questions about perceptions of the police. Methodologically, the estimation of causal effects depends on two important assumptions: the excludability assumption (sometimes also referred to as the temporal stability assumption) simply implies that without the treatment event, there would be no effect on the outcome. Testing for this assumption therefore includes the checking of collateral events and unrelated time trends. The ignorability assumption states that the chance of being assigned to either pre- or post-event group should be as-good-as-random.

The current review has two aims:

1. to synthesize the impact of unexpected events on trust in police across different contexts and types of events, and
2. to evaluate the methodological characteristics of each study with attention to the assumptions for causal inference.

As a result, we expect to contribute to the understanding of the impact of vicarious experiences on trust in police among the general public, as well as a critical overview of the use of natural experiments in research on trust in police.

In addition, we will provide a thorough but accessible guide for implementing the UESD approach in criminological research, with an application to policing research. Our focus will be on clear identification of main effects. Therefore, we will only address heterogeneous effects briefly and focus on testing the pertinent causal inference assumptions. Notable combinations of the UESD with the classical Regression Discontinuity Design (RDD) or the Difference-in-Differences (DiD) design will be addressed as well as statistical power issues and the correct calculation of standard errors. Accordingly, our summary of the UESD approach in this specific context will provide best-practice recommendations for its application in these contexts and beyond.

### Unexpected events and perceptions of police

Research on high-profile events, such as police violence or terrorist attacks, tends to rely on two main theoretical frameworks to explain public response to these events. The first framework is concerned with explaining how perceptions might change in response to specific police actions during incidents. Based on procedural justice theory, the public formulates their judgements based on how the police treated those involved in the incident (Tyler 2006; Tyler and Jackson 2014). When the police treat people with fairness, respect, and neutrality, the public is expected to revise their views of the police upwards, leading to higher trust and legitimacy (Hough et al. 2010; Sunshine and Tyler 2003). Incidents of police violence can be seen to violate these principles of procedural justice, resulting in more negative perceptions of police (Curtice 2021; Nägel and Lutter 2021; Perry et al. 2017; White et al. 2018). Research shows that following high-profile incidents of police violence, public reactions can be polarized, whereby members of affected minority groups may react more strongly than majority groups (Kochel 2019; Nägel and Lutter 2021; Reny and Newman 2021). This also relates to distributive justice theory since police violence against ethnic minorities can trigger the notion that misconduct is unevenly distributed among ethnic groups (Weitzer 2019).

The second framework aims to explain changes in attitudes following major crises, such as terrorist attacks or public health emergencies. Based on the notion that these types of events can be characterized as an “external” threat to societal institutions and daily life, the public is expected to “rally around the flag” to support threatened institutions, including the police (Perrin and Smolek 2009; Sibley et al. 2020; Van Hauwaert and Huber 2020). Collective threats that lead to uncertainty and angst are expected to “activate” in-group solidarity and diffuse support for political and criminal justice institutions (Porat et al. 2019; Schraff 2020; Sibley et al. 2020). From this perspective, rally effects are considered emotional reactions to collective threats as opposed to post hoc evaluations of institutional response to crises (Schraff 2020). For example, “rally effects” were observed following the onset of the COVID-19 pandemic and subsequent nationwide lockdowns in March 2020 (Bol et al. 2021; Esaiasson et al. 2021; Kritzinger et al. 2021; Schraff 2020). The COVID-19 pandemic has been described as a “foreign” or external shock “inflicting sudden and unprecedented hardship on societies at large” (Kritzinger et al. 2021, p. 1206).

Given that events occur unexpectedly during survey fieldwork, the temporal window before and after treatment can vary substantially across countries. While some studies have found lasting “rally effects” on support for political institutions (e.g., several weeks to over a year, Gaines 2002; Hetherington and Nelson 2003), changes in trust in police following police actions or societal crises tend to be short-lived, sometimes lasting only days or weeks following the event (Nägel and Lutter 2021; Reny and Newman 2021).

### Issues with causal inference: ignorability and excludability

Researchers have called attention to the lack of causal evidence to support the connection between police actions, public opinion, and behavior (Graziano 2019; Nagin and Telep 2020). As a quasi-experiment, the UESD allows the dynamics of public attitudes to be explored in a “natural setting” without sacrificing the unique advantages of random allocation that would be provided by an RCT. However, the natural setting also carries threats to validity, as the influence of unobserved variables, unknown seasonal time trends, and collateral events often cannot be completely ruled out or controlled for. This naturally places enormous demands on researchers to examine the relevant assumptions as rigorously as possible in order to utilize the promises of this identification strategy without falling into the many potential pitfalls. These important assumptions are referred to in contemporary research as the excludability assumption and the ignorability assumption (Muñoz et al. 2020).

Exludability, also known as the temporal stability assumption (Legewie 2013), implies that the timing, as an instrument for the focal event’s effect on the outcome, affects the outcome through no other channel than the event itself. This is conceptually similar to the exclusion restriction in instrumental variable estimation, since the exclusion restriction states that, conditional on covariates, the instrument (survey timing) cannot be correlated with the error term (e.g., other events) in the explanatory equation (Labrecque and Swanson 2018; Stock and Watson 2007). There are at least four situations in which this assumption might be violated: collateral events, simultaneous events, unrelated time trends, and endogenous timing of the event (Muñoz et al. 2020). Collateral events are happenings that are triggered by the actual event in question. Unrelated time-trends refer to much observed seasonal opinion patterns like mood changes (Rosenthal and Wehr 1987, which could lead to biased treatment effects when these variables also correlate with the outcome. Finally, endogenous timing of the event refers to a situation in which the focal event is triggered by actors who can strategically choose when to disclose information on said event. For example, when partisan media postpones printing articles on corruption scandals until close to an election to maximize potential damage, it becomes more difficult to isolate the event’s effect since public opinion might be driven instead by the election (Ares and Hernández 2017. Strategies to assess these potential violations include placebo treatments, inspection of pre-existing time-trends, falsification tests on other outcomes or other surveys/observations, as well as a detailed qualitative description of the event to give an overview of how the event rose in salience (Mellon 2014; Muñoz et al. 2020).

The ignorability assumption addresses whether the quasi-experimental setting can be considered as truly experimental, or, in other words, whether the chance of being assigned to either the control or the treatment group is as-good-as-random (Bor et al. 2014; Gangl 2010; Legewie 2013; Muñoz et al. 2020). Regarding this causal inference assumption, the reachability and regional sampling biases are concerned with potential systematic differences in survey participation (Legewie 2013). Muñoz et al. (2020) argue that fieldwork organization and attrition are also potential threats to the violation of this assumption. These violations result in imbalances between the pre- and post-event groups. The most straightforward test is a simple mean comparison of relevant covariates (i.e., age, education, sex, income) on the binary event indicator. While significant differences violate the ignorability assumption, there are strategies to address these violations and check robustness through covariate adjustment, matching procedures, and the use of multiple bandwidths. Non-response patterns can be analyzed to assess whether fieldwork determinants or attrition is a danger to the validity of the design. Additionally, a qualitative description of the event can provide insights into the extent to which the event was unexpected (Muñoz et al. 2020). If the event was foreseeable, participants may have changed their opinions beforehand and the timing of the interview becomes less reliable as an instrument for treatment assignment.^1^

## Methods

The purpose of this systematic review is to provide an overview of research that has examined the impact of unexpected events on trust in the police. The protocol for this systematic review was pre-registered on OSF in August 2021 [https://osf.io/4mj89/].

### Search strategy and criteria

#### Types of studies

This review focuses on studies that quantitatively assessed the impact of unexpected events on public perceptions of police. The events included in the review should have taken place during periods of ongoing survey fieldwork. This excludes studies that examined planned or reasonably foreseeable events such as the implementation of policies and more straightforward before and after designs (e.g., Kang 2021; Vidales et al. 2009).

#### Types of outcome measures

The outcome variable of interest is any attitudinal measure of general police performance, including confidence, trust, or overall satisfaction. This excludes perceptions measures of officer-reported perceptions of public approval (Turchan 2021), positive or negative sentiments scraped from Twitter (Oglesby-Neal et al. 2019), proximal behavioral responses such as cooperation with the police (LaFree and Adamczyk 2017), and emergency or service calls to the police (Zoorob 2020).

#### Additional inclusion criteria

As the focus is on causal inference using unexpected events, we did not restrict the type of event or the geographical origin of the data. The timeframe covered spans from 1970 to the date of the search (August 2021). The language of the study is restricted to English.

#### Search methods

The search strategy proceeded in three stages:

1. An initial search was performed using single database (Web of Science) in order to refine the search strings. Titles and abstracts were reviewed in order to add or adjust keywords. The initial search revealed that the use of methodological search terms such as “natural experiment” or “difference-in-differences” were too restrictive, and so they were omitted from the primary search.
2. The primary search was then conducted using the following keywords: “misconduct” OR “high-profile” OR “terrorist attack” OR “scandal” OR “corruption” OR “incidents,” police OR policing (alternatively, “trust in institutions” OR “trust in government”), and attitudes OR perceptions OR trust OR confidence.
3. Google Scholar was used to supplement the search of primary databases and to scan the citation lists to identify any studies that may have been missed.

#### Electronic databases

The primary search was conducted using three primary scholarly databases: Scopus, Web of Science, and EBSCO Host. Google Scholar was used to scan for pre-prints or other grey literature that may not be published in an academic journal or formal publication. In addition, the reference lists from the selected studies were also searched for any additional studies that meet the search criteria.

### Data collection and analysis

#### Study selection

The process to determine relevant articles for inclusion was conducted in two stages. First, titles and abstracts were reviewed and marked for inclusion or exclusion based on the search criteria outlined above. In cases where it was not possible to make a decision based on the abstract, the study was included for full-text screening in the next stage. Following general recommendations regarding the review of abstracts (Stoll et al. 2019; Waffenschmidt et al. 2019), all titles and abstracts were reviewed by both authors. In the second stage, full texts for all studies included were reviewed for eligibility by both authors. Any uncertain cases were resolved through discussion between the authors.

#### Coding scheme

All studies that meet the eligibility criteria were coded based on key study characteristics, methodological strategy, measurement, and effect sizes, including sign and significance. Each paper was coded by both authors, and any disagreements were resolved through discussion. In regard to methodological strategy, we coded the following characteristics: design (e.g., difference-in-differences, propensity score matching, regression discontinuity, OLS), assumptions addressed (excludability, ignorability), sample size (control, treatment), and temporal window (in days). Regarding the assumptions addressed, we evaluated the extent to which studies checked threats to the excludability and ignorability assumptions. Following the recommended practices by Muñoz et al. (2020), we evaluated the ignorability assumption based on whether or not researchers conducted balance tests, assessed multiple bandwidths, adjusted covariates for imbalances, and examined non-response patterns. We evaluated the excludability assumption based on whether or not they estimated placebo treatment effects, assessed pre-existing time-trends, and conducted falsification tests on other units and on other outcomes. In addition, we examined to what extent they provided an in-depth description of the event. The full coding scheme is available in the Online Appendix.

#### Data synthesis

Given the differences across event type and hypothesized effects, we opted for a narrative review of studies’ results and methodological characteristics. The synthesis is divided into two parts: first, we describe the types of events, hypothesized effects on trust in police, and the methodological characteristics of each study including an evaluation as to what extent the key assumptions (ignorability, excludability) are addressed. Second, we summarize the findings of each study.

#### Deviation from the protocol

It was our primary intention to exclude studies with “procedural justice” as an outcome (see pre-registration). Given the low n of studies, and the conceptual similarities of procedural justice and trust/trustworthiness/confidence, as well as the comments by the peer reviewers, we decided to deviate from the protocol and include studies using procedural justice (i.e., Curtice 2021).

## Results

**Fig. 1:**
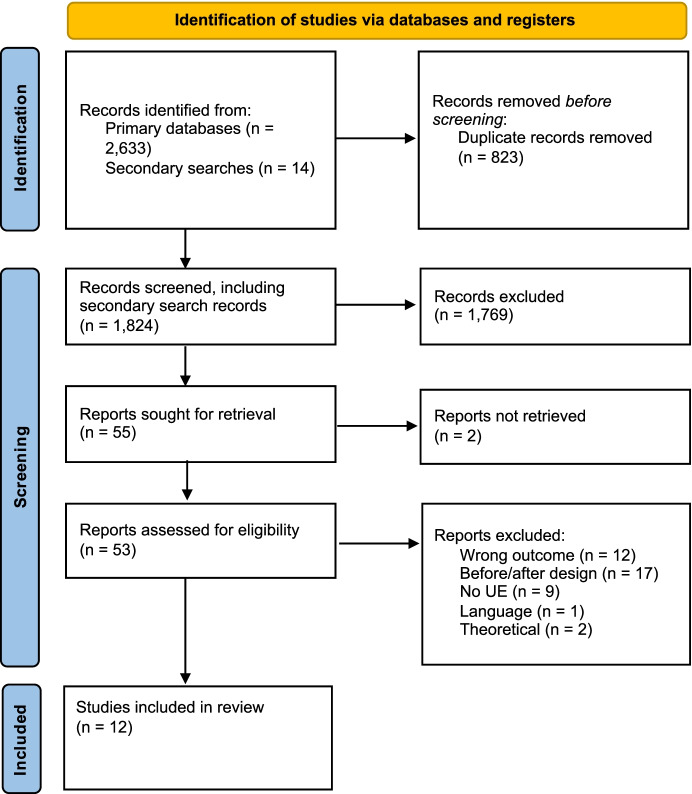
PRISMA flowchart showing the search and identification of studies

The search of databases resulted in 2,633 studies. Figure 1 shows the search and identification process in a PRISMA flowchart (see Page et al. 2021). Following the removal of duplicates (n=823), the abstracts of the remaining 1,810 studies were screened by both authors based on the inclusion criteria. A total of 55 potential studies were retrieved for full-text screening (n=41 from database searches, n=14 from the search of reference lists and citations). Next, the texts were reviewed to assess eligibility. The three main reasons for exclusion were that the study utilized a before and after design (n=17), the outcome was not attitudinal or did not measure trust/confidence in the police or procedural justice (n=12), and the study did not contain an unexpected event (n=9). The final sample included 12 independent studies.

### Characteristics of the included studies

Of the 12 identified studies that assess an unexpected event during survey design, half (n=6) were published in the last two years (since 2019) and the oldest was published in 2012. The geographical setting of the studies is relatively diverse, including the United States (n=2), United Kingdom (n=2), Spain (n=1), France (n=1), Russia (n=1), New Zealand (n=1), Zimbabwe (n=1), Uganda (n=1), and Afghanistan (n=1). Only one study took a cross-national approach, assessing unexpected events (protests) across 13 African countries. A summary of the included studies, events, and results is presented in Table 1.

#### Types of events and hypothesized effects

The types of events can be grouped into three broad categories (see Table 1). The first group of events capture incidents of police violence (n=6), typically as a stand-alone high-profile event (e.g., violence against or death of a minority group member, political repression). In two studies, the focus of the event was on mass mobilization or disorder that followed high-profile incidents of police violence (i.e., the 2012 London disorder following the death of Mark Duggan, the 2020 protests following the death of George Floyd). In all but one of these studies, the authors hypothesized that experiences of police violence would result in more negative perceptions of the police. One study did not provide a hypothesis (Hohl et al. 2013). Conversely, one study assessed the impact of peaceful protest policing following contested government elections (Frye and Borisova 2019). The expectation was that the use of peaceful instead of repressive treatment of protesters would lead to more trust in police.

The second group of events concern external threats or crises, most notably terrorist attacks (n=3) and the COVID-19 pandemic and lockdown (n=1). The terrorist incidents include the 2012 Taliban attack on a hotel in Kabul, Afghanistan, the 2004 Madrid train bombings, and a variety of domestic (UK) and international (Continental European) terrorist attacks. For both the terrorist and pandemic events, the authors hypothesized that trust in police would increase in line with “rally-round-the-flag” effects.

Finally, two studies examined events that do not fit well into the previous two categories. One study examined the effect of political protests on trust in police, arguing that protests signal to citizens that state institutions are not doing their job monitoring political actors or providing services (Sangnier and Zylberberg 2017). Another study examined the impact of incidents of political repression on trust in institutions, including a measure of trust in police (García-Ponce and Pasquale 2015). However, they hypothesize that this will lead to higher trust in police because respondents fear political reprisal.

**Table 1:** Summary of study characteristics and results

Author(s)	Year	Location	Unexpected event	Data source	Hypothesized effects (mechanism)
Curtice	2021	Uganda	Violent political repression	Twaweza/Ipsos	less procedural justice (legitimacy)
Deglow & Sunderberg	2021	Kabul, Afghanistan	Terrorist attack on hotel	Survey of the Afghan People	more trust (rally), less trust (performance)
Dinesen & Jaeger	2013	Spain	2004 Madrid train bombing	Eurobarometer	more trust (rally)
Fenn & Brunton-Smith	2021	London, UK	Domestic and international terrorist attacks	Metropolitan Police Attitudinal Survey	more trust (rally), less trust (performance)
Frye & Borisova	2019	Moscow, Russia	Allowing (peaceful) government protests	Authors’ own survey	more trust (legitimacy)
Garcia-Ponce & Pasquale	2015	Zimbabwe	Violent political repression	Afrobarometer	more trust (falsification)
Hohl et al.	2012	London, UK	2011 London disorder	Metropolitan Police Attitudinal Survey	less trust (legitimacy)
Naegel & Lutter	2021	France	Police violence against black man in Paris, subsequent riots	European Social Survey	less trust (legitimacy)
Reny & Newman	2021	USA	Protest police violence (death of George Floyd)	Nationscape Survey	less trust (legitimacy), null (long-term)
Sangnier & Zylberberg	2017	13 African countries	Government protests	Afrobarometer	less trust (performance)
Sibley et al.	2020	New Zealand	Covid-19 lockdown	New Zealand Attitudes and Values Survey	more trust (rally)
White et al.	2018	Baltimore, USA	Police violence (death of Freddie Gray)	National Institute on Drug Abuse survey	less trust (legitimacy), null (long-term)

The mechanism of the hypothesized effect(s) are reported in parentheses. “Rally” refers to rally-round-the-flag effects, “performance” refers to judgements based on police performance, “legitimacy” refers to updating mechanisms based on police procedural and distributive justice, “long-term” refers to the notion that attitudes are developed early and stable long-term, and “falisfication” refers to falsified responses based on fear. NR=not reported

### Methodological characteristics and assessment

#### Research design

While all studies examined unexpected events that occurred during survey fieldwork, they adopted a wide range of designs and analytical methods to answer their research questions (see Table 2). The majority utilized some form of linear or ordered logit regression (n=6), sometimes as an additional analysis alongside either regression discontinuity designs [RDD] or difference-in-differences [DiD] designs (n=2).^2^ Two studies utilized propensity score matching in combination with paired sample t-tests to estimate the mean differences in trust in police between pre- and post-event groups (n=2). One study aggregated responses by day and estimated the impact of the event using interrupted time series analyses [ITS]. In one study, the proportion of responses in a given category was compared before and after the event (Hohl et al. 2013). However, it is not entirely clear what type of analysis or method was used to estimate differences.

#### Sample characteristics

One important methodological characteristic is the size of the sample for the “treatment” (post-event) and “control” (pre-event) groups. In a UESD study the size of these groups is dependent on the timing of the event during fieldwork. As such, if the event occurs very early or late during fieldwork, the difference in the size of the groups may be large, which can result in methodological issues that need to be addressed (e.g., power, heteroskedasticity). This is the case in the study analyzing the impact of the Madrid train bombings (Dinesen and Jaeger 2013), wherein the event occurred 7 days before the completion of the Eurobarometer fieldwork. As a result, the treatment sample consisted of 72 respondents. The size of these groups may also depend on the chosen design. For example, the selection of an optimal bandwidth (e.g., by a mean square error [MSE] approach) in RDDs can restrict the analytical sample to only a small number of days before and after the event (e.g., 6 days, n=130 in Nägel and Lutter 2021). Those using RDD therefore typically assess multiple bandwidths to ensure robust results (Stommes et al. 2021). Both papers applying a RDD in this review are using a non-parametric automated MSE bandwidth selection method.

**Table 2:** Summary of sample characteristics and temporal window used to assess treatment

Author(s)	Control N	Treatment N	Design	Temporal window post-event (days)
Curtice	1789	131	Regression	9
Deglow & Sunderberg	401	318	Regression	6
Dinesen & Jaeger	928	72	Regression	7
Fenn & Brunton-Smith	NR	NR	ITS	182-2445(a)
Frye & Borisova	611	939	Regression	15
Garcia-Ponce & Pasquale	NR	NR	Regression	10
Hohl et al.	NR	NR	NR	53 (b)
Naegel & Lutter	1853	207	RDD	3,6,13
			Regression	37
Reny & Newman	NR	NR	RDD	7
			Regression	98
Sangnier & Zylberberg	NR	NR	DiD	60
Sibley et al.	1003	1003	PSM+t-test	18
White et al.	259	1269	PSM+t-test	134(b)

NR=not reported; Regression=OLS, logit, or probit regression; ITS=interrupted time series; RDD=regression discontinuity design, bandwidth reported for temporal window; DiD=difference-in-differences; PSM=propensity score matching

$^{\mathrm {a}}$ Multiple events analyzed, so N and temporal windows varied depending on event date. Range calculated based on dates of events and fieldwork provided in the study;

$^{\mathrm {b}}$ Temporal window calculated based on the date of the event and fieldwork provided in the study

Two studies did not report the sample size for control and treatment groups (Hohl et al. 2013; Reny and Newman 2021). Two studies used events with varying timing and location (violent political repression, protests), which implies variation in the size of treatment and control groups per event (García-Ponce and Pasquale 2015; Sangnier and Zylberberg 2017). However, in both of these studies the size or average size of each treatment and control group was not reported. In three studies, the size of the treatment and control groups were relatively balanced, with one study using 1-to-1 matching to achieve balanced pre-and post-event groups. The remaining studies reported relatively large differences in the size of treatment and control groups.

#### Assumptions addressed

Table 3 provides an overview of the extent to which the included studies assessed or addressed the excludability and ignorability assumption. As has been described above, there are different strategies to test or control for possible violations. A “1” in Table 3 indicates that the respective study has performed this robustness analysis, whereas a “0” indicates that this specific test/check is missing from that paper. Both authors coded the included studies independently. A comparison of the coding results showed an overlap of 95.45% between both coders. Deviating assessments were discussed accompanied by a re-assessment of a particular test/check in the respective study to find a solution. Finally, a score was computed reflecting an additive index of all possible robustness checks ranging from 0 to 9. This score can be interpreted as the respective study’s rigorousness in terms of the specific UESD assumptions.

**Table 3:** Evaluation of ignorability and excludability assumptions

	Ignorability				Excludability				Both	
Author(s)	Balance tests	Multiple bandwidths	Covariate adjustment	Non-response	Placebo treatments	Pre-existing time trends	Fals. tests (other units)	Fals. tests (other outcomes)	Event description	Score
Curtice	1	0	1	0	1	0	0	1	1	5.00
Deglow & Sunderberg	1	1	1	1	1	1	1	0	1	8.00
Dinesen & Jaeger	1	1	1	0	0	0	0	0	1	4.00
Fenn & Brunton-Smith	1	0	1	0	0	1	0	0	1	4.00
Frye & Borisova	1	1	1	1	0	0	1	1	1	7.00
Garcia-Ponce & Pasquale	1	1	1	1	0	0	0	0	1	5.00
Hohl et al.	0	0	0	0	0	0	0	0	0	0.00
Naegel & Lutter	1	1	1	0	1	0	1	1	1	7.00
Reny & Newman	1	1	1	1	1	1	0	1	1	8.00
Sangnier & Zylberberg	1	1	1	1	0	1	1	0	1	7.00
Sibley et al.	1	0	1	0	0	0	0	0	1	3.00
White et al.	1	1	1	0	0	1	0	0	1	5.00
Count column (out of 11)	11	8	11	5	4	5	4	4	11	5.25

1=Test checked/applied; 0=Test not checked/applied; Fals.=falsification

Eleven out of twelve studies used balance tests, multiple bandwidths, and provided an in-depth description of the focal event. Most (n=8) have extended a naïve before/after estimation by applying a model using control variables or matching procedures. Less than half of the included studies analyzed non-response patterns and/or pre-existing time-trends (n=5). Four studies examined alternative observation units by estimating results using the same survey at the same time in other countries, and/or the same survey at a different time in the same country. Finally, only three investigated placebo treatments and falsification tests on other outcomes, namely by assessing effects on variables very similar to the actual outcomes that should theoretically not be affected by the treatment.

It is clear from Table 3 that the ignorability assumption, with the exception of the analysis of non-response patterns, is subjected to much more rigorous scrutiny than the excludability assumption. Out of 60 possible tests of the ignorability assumption 46 (77%) were performed, while out of 60 possible tests of the excludability assumption only 27 (45%) were performed.

#### Summary of effects

Given that the first two groups generally share similar theoretical frameworks and hypotheses, we summarize the effects of studies according to the categories discussed above (for summary of results, see Table 4). Two out of six studies examining the effects of police violence did not find significant effects on trust in police (London disorder, Hohl et al. 2013; the death of Freddie Gray, White et al. 2018). Three of the remaining four studies found that incidents of police violence lead to significantly less trust in police (violence against Theo L. in Paris, Nägel and Lutter 2021; death of George Floyd, Reny and Newman 2021; violent repression by police in Uganda, Curtice 2021), and the fourth found that peaceful protest policing was associated with increases in trust in police (peaceful protests in Russia, Frye and Borisova 2019). All four studies examining external threats or crises found to some extent significant “rally effects” on trust in police (attack on hotel in Kabul, Deglow and Sundberg 2021; Madrid train bombings, Dinesen and Jaeger 2013; various domestic and international attacks, Fenn and Brunton-Smith 2021; COVID-19 lockdown in New Zealand, Sibley et al. 2020). One study assessed a wide range of domestic and international terrorist attacks that occurred within a particular time frame (2011–2018, Fenn and Brunton-Smith 2021). When all domestic and international attacks are included in the model, the effect of terrorist attacks leads to increases in support for the police after only three out of the five domestic (UK) Islamic incidents.

The remaining two studies both found effects consistent with their hypotheses. Protest incidents across 13 African countries were associated with significantly less trust in police (Sangnier and Zylberberg 2017). Violent political repression in Zimbabwe was associated with increases in trust in police, which was explained by the respondents’ fear of political consequences from the Mugabe regime (García-Ponce and Pasquale 2015).

**Table 4:** Summary of effects

Author(s)	Unexpected event	Result	Effect size significance
Curtice	Violent political repression	less procedural justice	sig
Deglow & Sunderberg	Terrorist attack on hotel	more trust	sig
Dinesen & Jaeger	2004 Madrid train bombing	more trust	sig
Fenn & Brunton-Smith	Domestic and international terrorist attacks	more trust (Islamic), less trust (right)	partial
Frye & Borisova	Allowing (peaceful) government protests	more trust	sig
Garcia-Ponce & Pasquale	Violent political repression	more trust	sig
Hohl et al.	2011 London disorder	null	ns
Naegel & Lutter	Police violence against black man in Paris, subsequent riots	less trust	sig
Reny & Newman	Protest police violence (death of George Floyd)	more negative views	sig
Sangnier & Zylberberg	Government protests	less trust	sig
Sibley et al.	Covid-19 lockdown	more trust	sig
White et al.	Police violence (death of Freddie Gray)	less trust	ns

ns=not significant; sig=significant at 0.05 level; partial=not all incidents significant

## Discussion

Given the number of ongoing large-scale surveys across countries, it is likely that researchers will increasingly take advantage of the UESD approach to assess the causal impact of relevant high-profile events on criminological outcomes. The current systematic review focused on studies that utilized UESD to evaluate the impact of events on trust in police, and the methodological findings are informative for any researchers interested in applying UESD to assess causal effects on an outcome of interest. Specifically, we highlight four important findings broadly concerning the substantive effects of events on trust in police, issues related to design and power, and the evaluation of assumptions to detect threats to internal validity.

First, our synthesis found evidence that public trust in police does change following high-profile events, but these effects are not consistent across events and contexts. Three out of five studies that evaluated incidents of police violence found significant negative effects on trust, whereas three out of four that evaluated major crises (e.g., terrorist attacks) found positive “rally” effects on trust in police. While the current narrative review approach does not allow us to quantitatively examine which factors may account for these mixed results, one possible explanation may be due to the prior level of institutional trust and contextual setting in which the event takes place. In relation to legitimacy mechanisms, some policing researchers have noted that in order to affect and observe change in trust in police, levels of trust among the public must first be at a level amenable to change (White et al. 2018). White et al. (2018) for example suggested that their null results on trust following the death of Freddie Gray may be because levels of legitimacy and trust in police in Baltimore were already low resulting in a “floor effect” whereby public opinion could not decline much further. Other research in “high-trust” societies has found that negative policing events were not associated with substantive changes in trust (Kääriäinen et al. 2016; Thomassen et al. 2014). The authors in these studies suggest this may be due to already high “reserves” of trust in police prior to the events. More research is needed to understand how societal and institutional characteristics may shape public response to high-profile, vicarious incidents involving the police.

The “asymmetry hypothesis,” which is rooted in theories of negativity bias (Rozin and Royzman 2001), suggests that negative experiences with the police trigger stronger public reactions than positive experiences with the police (Skogan 2006). While there is evidence to support asymmetry on the basis of personal encounters and media effects (Choi 2021; Li et al. 2016), our review found that both positive (peaceful protest policing) and negative (police violence, repression) experiences influenced attitudes toward the police. However, we are not able to compare the relative size of the effect between positive and negative events. In addition, most studies on vicarious experiences in our review focused on negative events. Only one study assessed the effects of positive encounters, i.e., the “unexpected” peaceful policing of anti-government protests in Russia (Frye and Borisova 2019). It is therefore not yet possible to judge to what extent more positive “trust-generating” incidents influence public opinion (or not). Future research should assess more positive or neutral events in order to appropriately evaluate possible asymmetric effects of police actions or policies on public opinion.

Additionally, given the wide range of attention to assessing causal assumptions, it is difficult to draw conclusions about the causal impact of vicarious experiences or crises on attitudes toward police. However, the five studies that scored relatively high on checking assumptions ($\ge$ 7 out of 9) do find significant changes in trust in police following incidents of police (non) violence and protest (Frye and Borisova 2019; Nägel and Lutter 2021; Reny and Newman 2021), protests against the government (Sangnier and Zylberberg 2017), and a terrorist attack (Deglow and Sundberg 2021). With regard to the procedural justice framework, this provides some causal evidence that vicarious (positive and negative) experiences can shape public trust in police (Nagin and Telep 2020). However, it is unclear to what extent these effects are lasting (Nägel and Lutter 2021; Reny and Newman 2021).

Second, we found substantial heterogeneity in research designs across the twelve studies included in the review. This includes choices regarding analytical approach, whereby studies applied a wide range of tools, including difference-in-differences design, regression discontinuity design, OLS regression, and propensity score matching with paired-sample t-tests. While all of these analytical approaches are valid for estimating treatment effects, some are better equipped than others to assess and minimize threats to internal validity and therefore estimate causal treatment effects. For example, while propensity score matching and mean comparisons allow one to control for observed differences between groups, the validity of the results depends on the choice of covariates to be matched (Guo et al. 2020; King and Nielsen 2019).

When panel data are available, the difference-in-differences design can add confidence in the validity of the design because it is possible to control, for example, for seasonal patterns in the outcomes of interest. It is not entirely implausible that trust in the police may be influenced by certain annual events, for example, by the May Day Rallies against police in Germany^3^. Given a panel data set, it would be possible to operationalize the treatment group using a binary indicator (before and after each day of the event) independent of the survey year, while including a time dummy for the year. The treatment effect $\delta$ could then be modeled as an interaction effect in the form $\delta$ (Treatment${}_{it}$ Time${}_{t}$), thus controlling for seasonal time trends. This modeling strategy would allow one to estimate the difference-in-differences outlined above, while simultaneously controlling for covariates.

In a similar vein, the decentralized institutional organization of police forces in several countries (e.g., the USA, Germany) can be exploited to analyze the effect of exclusively locally relevant events while using other regions as control groups. For example, a specific policing event or policy change in one federal state might affect local attitudes within that state but not in all other federal states, which can be used as control groups in a DiD design. The effect of interest could then be modelled as the slope coefficient of the interaction between a before/after indicator and a dummy of the federal state of interest. The most relevant additional assumption in such a design is the parallel trends assumption which implies that if there had been no event or policy change in the federal state of interest, the mean difference between treatment and control group should have stayed unchanged in the post-treatment period as it was in the pre-treatment period (Huntington-Klein 2022). With cross-sectional data, the regression discontinuity approach can enhance internal validity because it allows for a quasi-random assignment between treatment and control group (Bor et al. 2014).

Recent comparisons showed that the regression discontinuity design is comparable to randomized controlled trials in terms of internal and external validity (Chaplin et al. 2018). As Stommes et al. 2021 discuss, this might still be conditional on having enough observations in the vicinity of the cut-point. Following their advice, we therefore recommend the use of the regression discontinuity design with automated bandwidth selection procedures and alongside a more traditional before/after setup. Given the limited bandwidth in the regression discontinuity analysis, a more straightforward regression approach would also allow one to examine the development of treatment effect by adding day or a week dummies step-by-step to estimate long-term vs. short-term effects.

Third, Table 2 shows rather large differences between treatment and control group which raises concerns about the equal distribution of residual variances. We recommend checking for issues of heteroscedasticity and the use of robust standard errors where applicable. As a general procedure, we suggest computing both heteroscedasticity robust and homoskedasticity-only robust standard errors and to report the more conservative ones (Angrist and Pischke 2009).

Relatedly, certain elements of the design, such as the size of the treatment and control groups, are often out of the researcher’s control since they are determined by the timing of the event during fieldwork. As has been described, our review showed that for some studies the size of the treatment and control groups were highly unbalanced. For example, in one study the event occurred 7 days before the end of survey fieldwork, resulting in n=72 for the treatment group (Dinesen and Jaeger 2013). Such differences in group size may result in insufficient power to detect an effect, particularly if researchers are interested in evaluating short- vs. long-term effects using different temporal windows. Since increasing the sample size is usually not possible, statistical power analyses such as sensitivity analysis can be useful to determine the minimum size of the effect that can be detected reliably given a fixed alpha, the available sample size, and desired power level (Perugini et al. 2018).

Post hoc or retrospective power analyses using observed effect sizes are not advisable, as the results can be misleading (Faul et al. 2007). Calculating the minimum detectable effect size allows the researcher (and reader) to judge to what extent the range of effect sizes can be considered realistic given existing knowledge (Perugini et al. 2018). Effect sizes that are larger than the minimum detectable value may occur due to chance, but also may be a signal that the result is upwardly biased (Lakens 2021). Sensitivity analyses can be computed easily using available power analysis programs such as G*Power (Faul et al. 2007). For example, a hypothetical survey in which the unexpected event occurred later during fieldwork may result in sample group sizes of n=500 in the control (pre-event) and n=50 (post-event). A sensitivity analysis specifying a desired power level of 0.80 and alpha of 0.05 for a (two-tailed) test of mean differences would be able to detect a minimum effect size of d=0.42. This means that for unbalanced groups with small sample sizes, the observed effect must be relatively large in order to be statistically significant (Lakens 2021).

Finally, our result shows that the ignorability assumption is addressed more often and more rigorously by the included studies than is the excludability assumption. In particular, studies often failed to evaluate placebo treatments and conduct falsification tests on other units and outcomes (see Table 3). The falsification checks for example are essential to rule out the possibility that the outcome might be affected by another event related to the timing of the fieldwork (Muñoz et al. 2020). Our advice in this instance is twofold: first, studies applying this particular research design should follow the good practice recommendations as closely as possible (see Legewie 2013; Muñoz et al. 2020). The reliability of the UESD can, where appropriate data are accessible, be improved by RD or DiD identification strategies. In this case, however, it is imperative that researchers adhere to the specifications of the relevant literature (Angrist and Pischke 2009; Skovron and Titiunik 2015), as the RDD in particular has shown to be prone to power issues, rendering findings either exaggerated or spurious (Stommes et al. 2021).

Second, journal editors and peer reviewers should carefully check to what extent researchers have systematically evaluated UESD assumptions, in particular concerning excludability. These assumptions are essential to check because there are many reasons why estimates can be biased in quasi-experimental designs that use observational data (Gangl 2010).

To summarize, the UESD approach is of particular interest for criminological contexts, as experimental manipulation of relevant stimuli is often ethically impossible. Additionally, there are many theoretical reasons why events could affect public perceptions of police differently — especially given the various geographical contexts of the included studies — underlining the need for continuous research that allows a comparative perspective. Based on this discussion, and to facilitate the rigorous application of the design within this strand of research, we provide a best-practice guide that summarizes our recommendations for the application of UESD regardless of context, as well as some general suggestions:

1. *Prioritize events.* The overlap of events with relevant surveys should be assessed first, as opposed to scanning relevant surveys for overlap with events. This can ensure that the feasibility of a study does not become more important than its relevance (see Deaton 2010 for a comparable discussion regarding natural experiments in general).
2. *Pre-register studies.* If possible, the study should be pre-registered to avoid hypothesizing after the results are known, i.e., “harking” (Kerr 1998).
3. *Conduct sensitivity power analyses.* Sensitivity power analyses should be conducted a priori to calculate the minimum effect size that is likely to be detected given the before/after sample sizes. This can inform researchers about whether their UESD setup is well suited to answer the specific research question.
4. *Use appropriate standard errors.* Heteroscedasticity might play a substantial role when before/after sample sizes and characteristics differ substantially. Accordingly, both homoscedasticity-only and heteroscedasticity robust standard errors should be computed while reporting the more conservative ones in the manuscript.
5. *Evaluate assumptions.* If possible, all tests proposed by Munoz et al. should be performed to evaluate the Ignorability and Excludability assumptions (see Table 3).
6. *Use alternative methods for robustness checks.* DiD or RDD extensions can be employed in the fashion outlined above but only alongside a more straightforward UESD approach to avoid model dependence.
7. *Consider potential heterogeneous treatment effects.* When there are potentially heterogenous effects that are not included, this could be a source of omitted variable bias. However, it is important to ensure that potential interactions are modelled from covariates that could theoretically not be outcome variables themselves (e.g., ethnicity, gender).

### Limitations

It is important to note that while the current study focused on the outcome trust in police to improve comparability, this meant that a number of other comparable studies with related outcomes were excluded from the analysis (Curtice and Behlendorf 2021; Oglesby-Neal et al. 2019; Turchan 2021). Similarly, papers that did not meet the inclusions criteria because they did not correspond to the definition of UESD by Muñoz et al. were also excluded. For example, Revkin 2022 provides an empirical examination on whether respondents adopt their attitudes toward the police after the extremely violent and lethal repression of anti-government demonstrations in Iraq, finding a decrease in perceptions of federal but not local police who did not participate as excessively in repressing protestors. However, the paper relies on an analysis of attitudinal differences between two survey waves, rather than an analysis of an event occurring within the fieldwork period of a single survey, making it impossible to assess multiple bandwidths, placebo treatments or pre-existing time trends. Researchers should continue to critically and systematically assess the validity of causal inferences in studies using observational data in order to highlight good practice for a given research question, design, and data type.

In addition, the UESD requires that the event should occur during fieldwork, whereas events are very likely to occur outside of fieldwork or between two waves of data collection (Kochel 2019). Although the UESD is better equipped to address threats to internal validity, such as collateral events and selection bias, events that occur between waves of data collection among the same participants have the advantage of assessing within-person changes controlling for stable between-person characteristics (see, e.g., Kochel 2019; LaFree and Adamczyk 2017). We recommend to adjust for unobserved unit-specific and time-specific confounders using a two-way-fixed-effects modeling strategy even though this does not represent a design-based estimation strategy (Imai and Kim 2021) that can yield similar confidence in the results as the straightforward UESD.

Another key problem in almost all UESD studies is the restriction to only capturing short-term effects since fieldwork periods seldom span more than a few months. Ongoing surveys like the British “Metropolitan Police Public Attitudes Survey” or the American “Nationscape Survey” might, however, provide a more long-term perspective.

Although all but one (Frye and Borisova 2019) of the included studies relate to generally “negative” events, it should be noted that the quasi-experimental stimuli employed in the UESD setup need not be a crisis moment. An unexpected election outcome, the sudden retirement of a police chief, or a successful law enforcement operation that is discussed in the media could relate to outcomes of interest. Accordingly, our sample provides only limited variation since even the events that are hypothesized to increase public perceptions of police are understood to be crisis moments.

We want to stress that only 25% of the included studies (Curtice 2021; Deglow and Sundberg 2021; Nägel and Lutter 2021) cited the Muñoz et al. paper, which for the first time outlined the UESD approach. While this raises the question whether the other included papers can be classified as UESD, we are confident that this classification is appropriate for several reasons: First, most of the papers were published prior to (Muñoz et al. 2020), making it impossible for the researchers to cite the paper. Second, we adhere closely to the selection criteria which considered survey-based studies with an unexpected event during the fieldwork period used as treatment, i.e., the definition of the UESD provided by Muñoz and colleagues. Lastly, Muñoz et al. cite one of the included studies (Dinesen and Jaeger 2013) as an example UESD, increasing our confidence that our classifications of studies are generally comparable.

### Conclusions

Increasingly, scholars have emphasized the need for caution in interpreting causal effects in policing research that relies on observational data (Cook 2015; Graziano 2019; MacCoun 2005). For example, in procedural justice research, Nagin and Telep (2017, p. 7) argued that in order to credibly establish causality, a “demonstration of an exogenous manipulation of actual behavior affecting perceptions of procedurally just treatment and perceptions of legitimacy and ultimately legal compliance is necessary.” On the one hand, the UESD can provide the solution to these issues provided that (a) fieldwork periods for adequate survey data, ideally including more refined survey instruments than a single trust indicator, overlap with relevant policing events, and (b) studies adhere to rigorous methodological requirements. Alongside other design-based approaches to causal inference, the UESD can help to rule out “third common causes” and investigate issues of reversed causality that are common not only to research on trust in police (Nagin and Telep 2017) but also to criminological and policing research in general (Dezember et al. 2021; Sampson 2010).

On the other hand, our review showed that there are a variety of potential biases that threaten the validity of research findings from UESD studies. This is because in natural experiments there is only limited control for confounding factors compared to randomized controlled trials. Our technical suggestions put forward in this paper should help to ensure that future research is conducted as rigorously as possible to obtain the full benefits of this research design. Furthermore, it is our ambition that the narrative synthesis of research findings will provide further theoretical insights that can inform future research to more adequately understand when, to what extent, and in which contexts high-profile events influence trust in and legitimacy of the police.

## Supplementary Information

Below is the link to the electronic supplementary material.

Supplementary file 1 (pdf 85 KB)

## Footnotes

1 Muñoz et al. (2020) also include heterogenous treatment effects and post treatment bias in their list of possible violations. Post treatment bias refers to a situation in which both the outcome as well as a potential moderator will be affected by the event. For example, a Jihadist terror attack that could theoretically affect attitudes toward refugees. The effect might be stronger for people identifying with right-wing parties, but the event could also influence identification with those parties. This creates a “bad control” problem from a causal inference perspective (Angrist and Pischke 2009) since the group before the event is not comparable anymore to the group after the event due to a potential ideological shift among participants. Since we do not assess moderation effects, we do not specifically assess this violation.

2 The DiD design is based on the idea of extending the simple before-after comparison of a treatment group by a before-after comparison with a control group. The difference at time 2 (after the intervention/event) between the treatment and the control group is subtracted from the difference between the treatment and control group at time 1 (before the intervention/event). With approximate equivalence of the groups, it is thus possible to control for unobserved factors that might influence the outcome in both the control and the treatment group. The RDD, on the other hand, exploits the fact that a continuous variable, in the case of the UESD this is the date, assigns observations to either control or treatment group. As a result, observations very close to the cut-off should be comparable to each other in the sense of a quasi-random assignment, and the average differences in the outcome should be attributable to the treatment, conditional on certain testable assumptions (Skovron and Titiunik 2015). A crucial advantage of the RDD compared to the standard before-after estimator is the possibility to determine non-arbitrary bandwidths (i.e., timeframes) to evaluate local effects (Imbens and Kalyanaraman 2012).

3 See: https://www.dw.com/en/may-day-rallies-police-protesters-clash-in-berlin-paris/a-57395625.
