# Supplementary material for: Unexpected events during survey design and trust in the police: a systematic review
Source: J Exp Criminol. 2022 Jun 15:1–27. Online ahead of print. doi: 10.1007/s11292-022-09508-y (PMC9198199; doi:10.1007/s11292-022-09508-y)
Supplement: Supplementary file 1 — (pdf 85 KB) [file 11292_2022_9508_MOESM1_ESM.pdf]

Online Appendix

Unexpected events during survey design and trust in the police: A systematic review

Coding Scheme

- Author(s):
- Article title:
- Journal title:
- Year:
- Country:
- Incident type : open description
- Incident date(s):
- Data source: survey data used to measure outcome
- Fieldwork dates: Dates fieldwork took place (during event)
- Outcome(s) measured:
- Expected effect: As hypothesized in paper
- Design: analytical method
- Method: analytical method
- Assumptions: excludability, temporal ignorability
  - Balance tests: y/n
  - Multiple bandwidths: y/n
  - Covariate adjustment: y/n
  - Non-response: y/n
  - Placebo treatments: y/n
  - Pre-existing time trends: y/n
  - Falsification tests (other units): y/n
  - Falsification tests (other outcomes): y/n
  - Description of event: y/n
- Sample size: control, treatment
- Temporal window: before and after (in days)
- Effect size description: direction, significance
